# Neuro-oncological research output in Africa: a scoping review of primary brain tumors

**DOI:** 10.1007/s10072-023-07272-z

**Published:** 2023-12-26

**Authors:** Mostafa Hossam El din Moawad, Mohammad Al-Jafari, Amira Mohamed Taha, Jenan Walid A’amar, Omar Alsayed, Taha Fayad, Mohammed Ahmed Sadeq, Khaled Albakri, Ibrahim Serag

**Affiliations:** 1 Faculty of Pharmacy Clinical Department, Alexandria University, Alexandria, Egypt; 2 Faculty of Medicine, Suez Canal University, Ismailia, Egypt; 3 Faculty of Medicine, Mutah University, Al-Karak, Jordan; 4 Faculty of Medicine, Fayoum University, Fayoum, Egypt; 5 Jordan Hospital, Amman, Jordan; 6 Faculty of Medicine, Tanta University, Tanta, Egypt; 7 Faculty of Oral and Dental Medicine, Sinai University, North Sinai, Egypt; 8 Faculty of Medicine, Misr University for Science and Technology, 6th of October City, Egypt; 9 Faculty of Medicine, The Hashemite University, Zarqa, Jordan; 10 Faculty of Medicine, Mansoura University, Mansoura, Egypt

**Keywords:** Cancer, Research output, Africa, Neurology, Neuro-oncology

## Abstract

**Background:**

There is evidence that individuals of African ancestry, particularly those residing in Africa, suffer from an unfortunate amount of under-representation in cancer research worldwide.

**Aim:**

We aimed to analyze current research output and potentially predict future trends in neuro-oncological research in Africa. Investigating deficits in the field will assist in identifying top-performing countries, which ones face challenges, and how to solve them. Therefore, targeted interventions can be applied to overcome these challenges.

**Methods:**

We conducted a systematic computer-based search on the following databases (PubMed, Scopus, Web of Science, and Embase) for research articles related to the neuro-oncological field in Africa. We aimed to retrieve any article published in the period between 1 January 2000 and 10 January 2023.

**Results:**

We included 200 eligible articles in our study. The output of neuro-oncological research has been increasing over the past two decades, peaking in 2019. Among the included articles, clinical practice issues constituted the majority (80%), while public health-related topics accounted for 20% of the publications. Regarding the type of neurological tumor, neuroblastoma was the most common, with 26 articles (13%), meningioma with 21 (10.5%), and glioma with 16 articles (8%).

**Conclusion:**

The interest in African neuro-oncological research is increasing. Hence, there is a need for ongoing efforts to address issues with clinical practice and public health related to neurological tumors in the continent. Future studies should concentrate on filling in knowledge gaps and investigating novel methods for neuro-oncological conditions that affect African populations in terms of prevention, diagnosis, treatment, and management strategies.

## Introduction

Research shows that individuals of African ancestry, particularly those residing in Africa, suffer from an unfortunate amount of under-representation in cancer research, including (clinical trials) worldwide [1]. The lack of neuro-oncology research focused on African populations results in knowledge gaps about disease patterns, treatment responses, and outcomes for these groups [2]. In addition to the growing African population and burden of cancer, we must take action to reverse this trend [3]. African populations bear a higher burden of cancer compared to general populations, and they are affected by cancer in forms that are more aggressive and challenging to manage [4]. Globally, a large population of Africans can trace their roots to Western Africa, and it is easily argued that data from this region can help improve the global health disparities. Considering these statistics and facts, African oncologists have the prerogative to lead the way in developing research to improve the health of the African population [5].

Brain tumors were once thought to be rare among the African population [6]. However, there is a shift in the perception of the African neuro-oncological disease burden among practitioners. However, due to the lack of organized nationwide and continent-wide databases of brain tumor incidence, epidemiological studies can be challenging [7]. No previous assessment of neuro-oncological research production originated from Africa in the first 20 years of this century. Hence, it is essential to analyze areas of strength and deficiency and give recommendations based on analyzed data.

Furthermore, this will allow us to advocate for increasing the quality, quantity, and diversity of published articles and encourage neuro-oncologists to participate more in academic research to pave the way for a healthier tomorrow for all African peoples and mend the medical and academic gap between the African continents and the rest of the world.

We aimed to analyze current research output and potentially predict future trends in neuro-oncological research in Africa by investigating deficits in the field. This study will help us identify which countries are performing well in this field, which ones face challenges, and how to solve them. Therefore, targeted interventions can be applied to overcome these challenges.

## Methods

### Literature search

When conducting our scoping review, we adhered to the Preferred Reporting Items for Systematic Reviews and Meta-Analyses for Scoping Reviews (PRISMA-SR) checklist. We conducted a systematic computer-based search on the following databases (PubMed, Scopus, Web of Science, and Embase) for research articles related to the neuro-oncological field in Africa. Articles published between 1 January 2000 and 10 January 2023 were retrieved (Table 1).

**Table 1:** Search strategy used to find eligible articles in the databases

Keywords	Search strategy
CNS, cancer, Africa	(oncol* OR malign* OR metasta* OR noma* OR carcin* OR cancer OR tumor OR neoplasm) AND (Cns OR brain OR neuro* OR cerebr* OR mening* OR spinal nervous) AND (“Algeria” or “Angola” or “Benin” or “Botswana” or “Burkina Faso” or “Burundi” or “Cameroon” or “Cape Verde” or “African” or “Chad” or “Djibouti” or “Congo” or “Egypt” or “Guinea” or “Eritrea” or “Eswatini” or “Ethiopia” or “Gabon” or “Gambia” or “Ghana” or “Guinea” or “Guinea-Bissau” or “Ivory Coast” or “Kenya” or “Lesotho” or “Liberia” or “Libya” or “Madagascar” or “Malawi” or “Mali” or “Mauritania” or “Mauritius” or “Morocco” or “Mozambique” or “Namibia” or “Niger” or “Nigeria” or “Congo” or “Reunion” or “Rwanda” or “Senegal” or “Sierra Leone” or “Somalia” or “South Africa” or “South Sudan” or “Sudan” or “Tanzania” or “Togo” or “Tunisia” or “Uganda” or “Western Sahara” or “Zambia” or “Zimbabwe” or “Africa”)

### Eligibility criteria for screening

The following inclusion criteria were developed: Articles published in the neuro-oncological field, articles published by African authors (whether first authors or corresponding authors known from the affiliation of authors), and articles that focused on the clinical field or public health issues in Africa. If articles did not meet any of these criteria, they were excluded. The study should be conducted in Africa. If not mentioned, we looked at the affiliation of the first or corresponding author to include the articles with African affiliations.

### Data extraction and subgrouping

Three authors extracted the data from selected papers independently using Microsoft Excel sheets. Studies were classified according to their type into primary, secondary, or others. Moreover, we classified studies according to their study design. Primary research included randomized controlled trials, retrospective and prospective cohort studies, case–control studies, cross-sectional studies, case reports and case studies, and cadaveric studies. Secondary studies included literature, scoping, and systematic reviews. Others included studies like commentaries, editor letters, and expert opinion papers.

Furthermore, we classified studies based on whether they discussed a public health issue or a clinical practice topic. Clinical practice topics included any study on the clinical field through management, surgical procedures, and other clinical interventions. On the other hand, public health research was related to the population’s overall health. Those included studies on epidemiology, patient education, health promotion, incidence rates, etc.

We also collected information on the type of intervention discussed in the papers: chemotherapy, surgery, radiotherapy, immune therapy, or other therapy. The type of neurological tumor was classified according to the 2021 World Health Organization (WHO) classification into central nervous system (CNS) tumors: meningioma, craniopharyngioma, glioma, medulloblastoma, astrocytoma, oligodendroglioma, peripheral nervous system tumor: neuroblastoma, neurofibroma, cartilage tumors: chondroma, and others [8]. We extracted information on whether the tumor behavior in the study was benign or malignant.

Regarding public health studies, we classified them into six categories: workforce, service delivery, information management, infrastructure, governance, and financing. We chose this classification based on the modified definitions of the Harvard Program in Global Surgery and Social Change’s (PGSSC) National Surgical, Anesthesia, and Obstetric Plan (NSOAP) health system domains [7, 9, 10].

Furthermore, our focus is on acquiring research studies within neuro-oncology. Subsequently, these studies are assessed for their relevance to clinical practice or public health issues. Finally, our inclusion criteria require that the selected studies be published within the African context. In cases where the study’s location is unspecified, we identify it as African primarily by the predominant African authorship, particularly the first and corresponding authors. We classified studies based on the collaboration type, including African authors, foreign authors, or a combination of African and foreign authors. We classified journals according to their nationality into African and non-African journals. The nationality of the first author was also extracted depending on affiliation and the main nationalities of all co-authors.

### Statistical analysis

Statistical analysis was carried out to determine frequencies and percentages of all categorical variables using SPSS V.26. We used Microsoft Excel to construct our figures and charts.

## Results

### Database search and screening

**Fig. 1:**
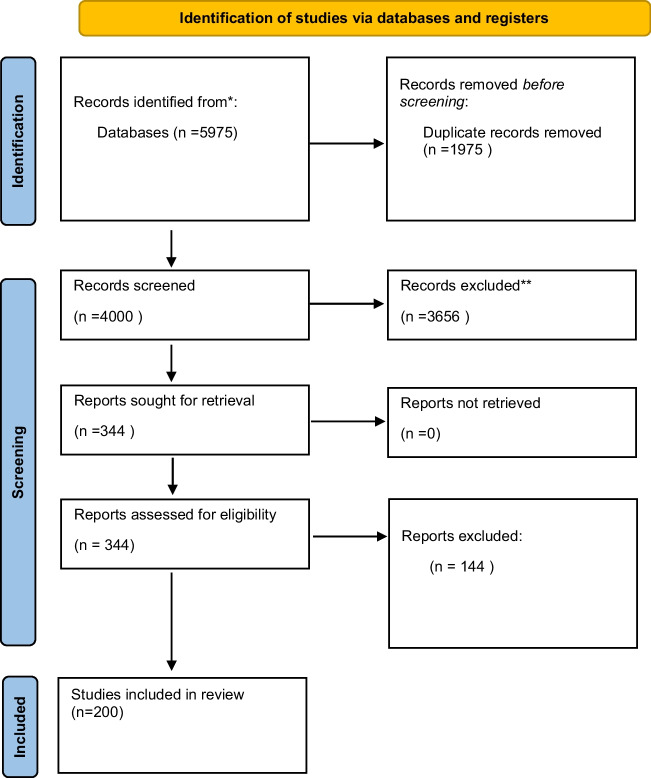
PRISMA-SR flow diagram of the included studies

Our search strategy yielded 5975 articles, which yielded 4000 after duplicate removal. Title and abstract screening resulted in 344 included articles; after full-text screening, 200 articles were eligible for the study (Fig. 1).

### Types of articles

Regarding types of research articles, 170 (85%) were primary research articles, 20 (10%) were secondary research articles, and 10 (5%) were classified as other types of research.

Among the primary research articles, the majority (39.5%) were found to be retrospective cohort studies, 29 (14.5%) case reports, and 29 (14.5%) prospective cohort. The types of research articles and their results are displayed in Table 2.

**Table 2:** Type of research articles and study designs

Types of research articles	*N* (%)
Primary	170 (85%)
Retrospective cohort	78 (39.5%)
Case reports	29 (14.5%)
Prospective cohort	29 (14.5%)
Cross-sectional	18 (9%)
Case series	8 (4%)
Randomized controlled trials	5 (2.5%)
Case–control	2 (1%)
Secondary	20 (10%)
Narrative review	9 (4.5%)
Systematic review and meta-analysis	8 (4%)
Scoping review	3 (1.5%)
Others	10 (5%)
Audits	2 (1%)
Letter to the editor	2 (1%)
Guidelines	2 (1%)
Book chapters	2 (1%)
Editorial	1 (0.5%)
Commentary	1 (0.5%)

### Timeline of the neuro-oncological research output in Africa

**Fig. 2:**
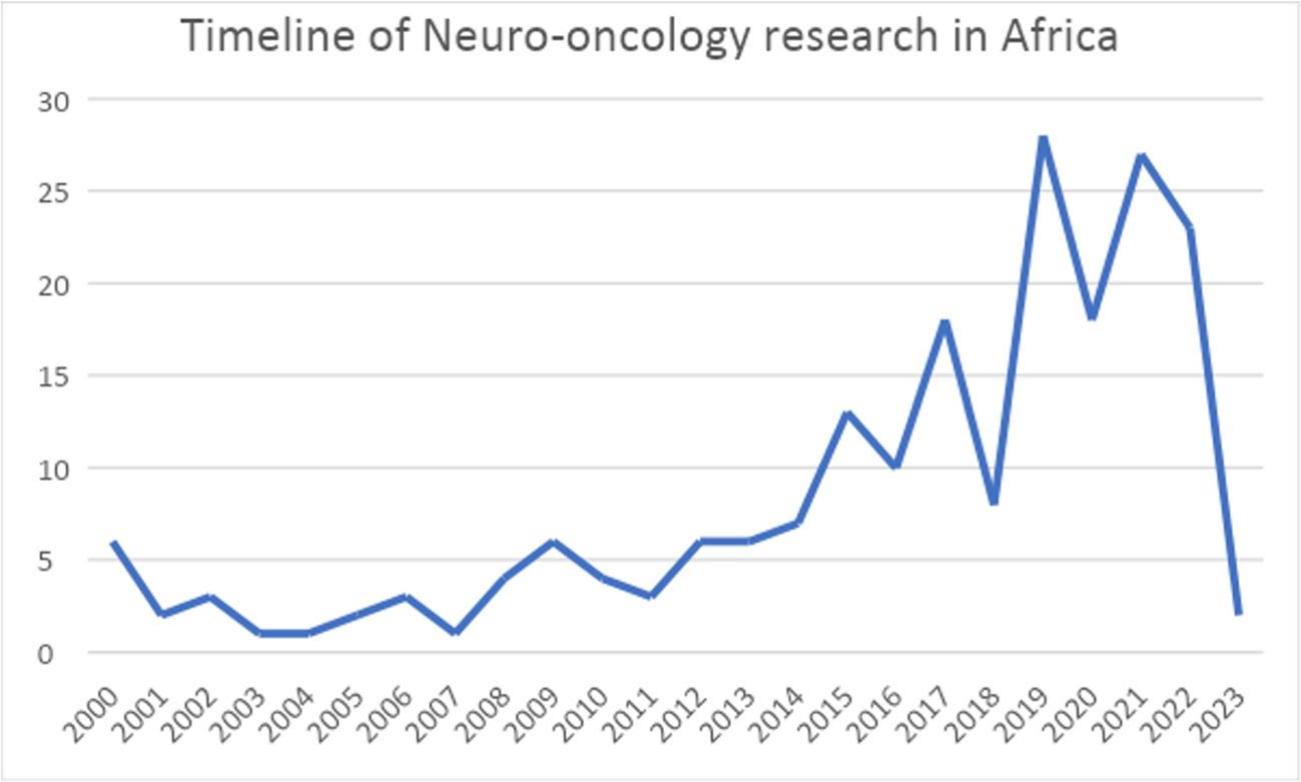
Timeline of neuro-oncological research output in Africa in the past two decades; 2023 is limited to January only

As shown in (Fig. 2), the output of neuro-oncological research has been increasing over the past two decades, with its peak in 2019 followed by 2021. Only two articles were published in 2023 until our database search.

### Authorship of the included studies

Most of the included articles were published by African authors only as they were reported to be 151 (75.5%) articles, followed by a collaboration between African and non-African authors, which were 37 (18.5%) articles, and foreign authors published 12 (6%) articles.

Nigerian authors were the most common among other nationalities, with a total of 44 (22%) articles, followed by Egyptian authors with 37 (18.5%), South Africa with 20 (10%), Morocco with 11 (5.5%), and Tunisia with 11 (5.5%).

### The framework of the published research

Regarding the scope of the published articles, the majority were articles discussing clinical practice issues, with 160 articles (80%). The remaining articles discussed public health, with 40 articles (20%). Regarding public health specialty, 13 (6.5%) were about the workforce, 10 (5%) articles were about information management, 8 (4%) articles were about service delivery, 6 (3%) were about finance, and 3 (1.5%) articles were about infrastructure.

Concerning clinical practice articles, we divided them into articles about chemotherapy, surgery, radiotherapy, immunotherapy, and other cancer-related fields.

The most published articles in clinical practice were about surgery, with 44 (22%) articles, 12 (6%) were about chemotherapy, 11 (5.5%) were about chemotherapy, surgery, and radiotherapy, 11 (5.5%) were about radiotherapy, 4 (2%) articles were about surgery and radiotherapy, 2 (1%) articles about immunotherapy, and 1 (0.5%) article about surgery and chemotherapy together. The rest, 75 articles (37.5%), were about other cancer-related fields**.**

Articles were mostly about malignant tumors, with 66 (33%) articles, followed by benign with 46 (23%) articles, and 10 (5%) articles about benign and malignant. The remaining articles (39%) did not report whether the tumor was malignant or benign.

Regarding the type of neurological tumor, neuroblastoma was the most common, with 26 (13%) articles, followed by meningioma with 21 (10.5%), and glioma with 16 (8%) articles. The percentage and number of the remaining neurological tumors are displayed in (Table 3).

**Table 3:** Types of most common neurological tumors in neuro-oncological research papers

Types of neurological tumors	*N* (%)
Neuroblastoma	26 (13%)
Meningioma	21 (10.5%)
Glioma	16 (8%)
Medulloblastoma	3 (1.5%)
Neurofibroma	3 (1.5%)
Craniopharyngioma	2 (1%)
Chondroma	1 (0.5%)
Several types of the mentioned ones in the same article	38 (19%)
Others	36 (18%)
Not reported	44 (22%)

We observed that about 38 (19%) articles investigated different neurological tumors, including meningioma, craniopharyngioma, astrocytoma, medulloblastoma, neurofibroma, glioma, and neuroblastoma. About 36 (18%) investigated other types of neurological tumors, and the type of neurological tumor was not reported in 44 (22%) articles (Table 3).

Regarding the age group of neurological tumors research articles, 84 (42%) were about adults, 60 (30%) were about children, 22 (11%) included both adults and children, and the remaining 34 (17%) articles did not report the age group.

### Journal nationality and most common journals

Regarding the nationality of the journal, 133 (66.5%) articles were published in a non-African journal, and 67 (33.5%) were published in African journals. The most common journal was “World Neurosurgery” impact factor (IF) = 2.23 with 19 (9.5%) articles, followed by “Child’s Nervous System” IF = 1.53 with 10 (5%) articles, followed by “Interdisciplinary Neurosurgery” IF = 0.18, “Journal of Neurosciences in rural practice” IF = 0.33 and “Pan African Medical Journal” IF = 0.28 with 5 (2.5%) articles for each, and “Journal of the Egyptian National Cancer Institute” IF = 0.33 with 4 (2%) articles (Table 4).

**Table 4:** The most common journals in which neuro-oncological African research is published in the past two decades

Journal	*N* (%)
*World Neurosurgery*	19 (9.5%)
*Child’s Nervous System*	10 (5%)
*Interdisciplinary Neurosurgery*	5 (2.5%)
*Journal of Neurosciences in Rural Practice*	5 (2.5%)
*Pan African Medical Journal*	5 (2.5%)
*Journal of the Egyptian National Cancer Institute*	4 (2%)

## Discussion

Our study showed that most articles about neuro-oncology published in Africa are primary studies, mainly retrospective cohorts. Most of the articles were published by African authors, with the peak of studies in 2019 rising. Clinical practice was the focus of researchers talking about surgery; the malignant type was predominant, and the type of neurological tumor most evident in research articles was neuroblastoma.

We acknowledge the difference in collaboration patterns between our study and the East African one [11]. Compared to the collaboration trend in East Africa, the predominance of articles authored solely by Africans in our research reflects the distinct nationalities contributing to our dataset—specifically, Nigerian, Egyptian, South African, Moroccan, and Tunisian researchers.

The number of neurosurgeons in Africa is considered minimal compared to Europe and USA. This results in decreased healthcare provided to neuro-oncological patients. The Nigerian Academy of Neurological Surgeons reported 81 neurosurgeons in Nigeria in June 2018. Compared to the average for sub-Saharan Africa, which is 1 neurosurgeon for every 2 million people, this translates to a neurosurgeon-to-resident ratio of 1 for every 2.4 million people [12, 13]. Europe has a ratio of 1 for every 100,000 inhabitants, as opposed to the USA, where it is 1 for every 62,500 [12]. A study published in 2016 found that the low number of neuroscientists is mainly caused by the technical and budgetary shortcomings in neurology training programs [14]. A lack of a defined policy on neurological illnesses and the unstable political environment in Africa has caused some of the continent’s neuroscientists to migrate to other continents.

Organizations like The Society of Neuroscientists of Africa (SONA), the International Brain Research Organization (IBRO), and national neuroscience societies offer domestic training for African neuroscientists [15]. Despite World Neurosurgical Association programs, challenges persist in prerequisites and facilities. Historically contributed to neurologist training by centers in Morocco, Egypt, Senegal, Tunisia, Nigeria, Ivory Coast, and South Africa, while NPOs collaborate for ongoing support and training in African universities [15].

Moreover, neurologists play a vital role in neurosurgeons in the neuro-oncology field, and as previously mentioned, a multidisciplinary team is required. However, there is also a shortage of neurologists in many African countries, such as South Sudan and Eritrea, with no neurologists, while Egypt (3108 neurologists) and South Africa (165 neurologists) have an adequate number of neurologists [16].

Our study identified 200 neuro-oncology articles published in African countries until January 2023. In comparison, a study in East African countries [11] found 36 articles by December 2020. Notably, our study revealed an article from Somalia, which the East African study reported as having none. Most of our articles were primary research, primarily retrospective cohort, echoing patterns observed in the East African study. Clinical care topics constituted 80% of our articles, aligning with the East African study’s emphasis on patient care (67%) [11].

Meningiomas are the only subtype of intracranial neoplasm, while gliomas are the most frequent subtype of primary parenchymal tumors, according to a large body of international research [11, 17]. Our study showed that the most mentioned type of neurological tumor in the research papers was neuroblastoma, followed by glioma and meningioma. However, the East African study showed that meningioma represents the largest proportion (43.3%) of common neurological tumors, followed by glioma (33.7%). This is like the report from the Central Brain Tumor Registry of the USA (CBTRUS), which showed that meningiomas made up 37.6% and gliomas made up 25.5% of all primary brain and other central nervous system tumors, respectively, in 2019 [18]. In the East African study [11], the majority of studies (47%) included adult and pediatric tumors, followed by pediatric only (31%) and then adult only (22%). However, our study showed that most were adult tumors, followed by pediatric, and then articles about both. Patients under 18 comprise half of the population in several East African areas.

Our study highlights most articles in non-African journals, indicating a limited presence in African institutes. The reported 65.2% absence of journal clubs aligns with challenges in fostering critical thinking [19]. Compared to the 85% inclusion in American schools, regular journal clubs enhance neurosurgery knowledge [20, 21].

In Africa, the limited availability of training slots (176) and neurosurgical programs (76) poses challenges for specialized medical training, especially in Sub-Saharan Africa, because the majority of programs are in North Africa [22]. While collaborative projects can enhance education and research, the scarcity of fellowship opportunities impedes professional advancement.

The study focuses on CNS tumor research in Africa, emphasizing the importance of research for clinical outcomes and global progress. However, limitations include potential oversight of studies conducted abroad by African neurosurgeons and the reliance on affiliations for location determination. The study calls for increased neurosurgery teaching, greater engagement of African neurosurgeons in research, and enhanced collaboration with foreign authors and institutions.

## Conclusion

This scoping review reveals an increasing interest in neuro-oncological research in Africa. The findings highlight the need for ongoing efforts to address issues with clinical practice and public health related to central nervous system tumors in the continent. Future studies should focus on filling the knowledge gaps and investigating novel methods for neuro-oncological conditions that affect African populations in terms of prevention, diagnosis, treatment, and management strategies.

## Data Availability

All generated data is included in this published article.
